# Intranasal Formaldehyde Exposure Induces RAGE-Mediated Alteration of the ADAM10/BACE1 Expression Balance and Amyloid Deposition

**DOI:** 10.3390/biomedicines14040779

**Published:** 2026-03-30

**Authors:** Ilya G. Mikhailov, Milana S. Mikhailova, Alexey D. Baklashov, Polina S. Ponamareva, Sofya N. Shumilova, Andrey N. Shuvaev, Olga S. Belozor, Anton N. Shuvaev

**Affiliations:** 1 Research Institute of Molecular Medicine and Pathobiochemistry, V.F. Voino-Yasenetsky Krasnoyarsk State Medical University, Partizan Zheleznyak Street 1, Krasnoyarsk 660022, Russia; 2 Institute of Biophysics, Federal Research Center “Krasnoyarsk Scientific Center of the Siberian Branch of the Russian Academy of Sciences”, Akademgorodok Street 50, Krasnoyarsk 660036, Russia; 3 Institute of Fundamental Biology and Biotechnology, Siberian Federal University, Svobodny Prospect 79, Krasnoyarsk 660041, Russia; brghtkey.0@gmail.com (A.D.B.); ashuvaev@sfu-kras.ru (A.N.S.)

**Keywords:** formaldehyde, Alzheimer’s disease, neuroinflammation, RAGE, amyloid deposition, ADAM10, BACE1, type 3 diabetes

## Abstract

**Background:** Alzheimer’s disease (AD) remains an incurable disorder with severe clinical consequences. The type 3 diabetes hypothesis posits that AD may constitute a neuroendocrine disorder driven by disrupted insulin and insulin-like growth factor signaling. Amyloid pathogenesis in AD is characterized by the accumulation of beta-amyloid (Aβ) monomers, their subsequent oligomerization, and amyloid deposition. One of the causes of Aβ accumulation is disruption of amyloid precursor protein (APP) processing due to imbalance in ADAM10 and BACE1 expression. In recent years, increasing attention has been devoted to investigating the role of environmental factors in AD pathogenesis. The receptor for advanced glycation end products (RAGE) serves as a key molecular link between environmental exposure and neuroinflammatory pathology. Formaldehyde (FA) is one of the most widespread environmental pollutants. Its involvement in amyloid plaque formation has been previously reported; however, the molecular mechanisms underlying this process remain insufficiently understood. Moreover, most available data are based on prolonged FA exposure, whereas industrial FA emissions are often short-term. The objective of this study was to determine whether brief intranasal administration of FA, modeling episodic industrial pollution, induces RAGE-mediated neuroinflammation and amyloid deposition in CD1 mice. **Methods:** Mice received intranasal FA at environmentally relevant 0.02 mg/day or 0.2 mg/day doses for seven days; an additional group was co-treated with insulin. Cognitive function was assessed using passive avoidance (PA) and radial arm maze (RAM) tests, and synaptic plasticity was evaluated by electrophysiology. Hippocampal tissue was analyzed for RAGE expression, *ADAM10*/*BACE1* gene balance, Aβ42 monomer levels, and amyloid deposits using optimized Thioflavin-S (Th-S) staining. **Results:** We observed cognitive decline in mice receiving intranasal FA administration. Elevated blood glucose levels were also observed following intranasal FA exposure. Sustained impairment of glucose metabolism led to overexpression of the RAGE in the hippocampus. There was also an imbalance of ADAM10 and BACE1 expression in the hippocampus. This was caused by overexpression of RAGE, as the enhanced interaction of the ligand and RAGE is a key factor disrupting this balance. Finally, Th-S staining confirmed amyloid deposition in mice subjected to intranasal FA exposure. **Conclusions:** This study provides new insights into the RAGE-mediated mechanisms by which FA contributes to the pathogenesis of AD.

## 1. Introduction

Dementia is characterized by a complex impairment of neuronal network functioning, which includes a decline in cognitive abilities, disturbances of memory, speech, and orientation. According to recent data, approximately 55 million people worldwide are currently living with dementia, with nearly 10 million new cases diagnosed each year. The World Health Organization estimates that by 2030 the number of people with dementia will increase to 78 million, and by 2050 to 139 million. AD is the most common form of dementia [1]. In 2005, the concept of type 3 diabetes was proposed as a hypothesis for the pathogenesis of AD. This condition is thought to arise from reduced expression and impaired signaling of insulin and insulin-like growth factors. This theory posits that AD may represent a form of neuroendocrine disease, sharing features with both type 1 diabetes (insulin deficiency) and type 2 diabetes (insulin resistance) [2]. Despite numerous studies investigating the relationship between AD and diabetes, the etiology and molecular mechanisms underlying the development of AD and other dementias remain insufficiently understood [2,3,4,5].

It has been noted that environmental factors play a significant role in the development of AD. The most common air pollutants include particulate matter, gases, organic compounds, and metals. Currently, concentrations of hazardous substances exceed maximum permissible concentration (MPC) in many cities of Russia [6]. Among them, FA is classified as a highly hazardous substance. Annual average concentrations of FA exceeding 1 MPC have been registered in 146 cities in Russia. Elevated atmospheric FA levels are also reported in cities across East, South, and Southeast Asia, Central Africa, North and South America [6,7,8,9,10,11]. Therefore, this issue is globally relevant.

The “lung-nose-brain” axis represents an important pathway through which inhaled environmental toxins may directly reach the central nervous system (CNS) [12]. Although the blood–brain barrier (BBB) provides substantial protection, the olfactory pathway constitutes a direct route from the nasal epithelium to the brain parenchyma, bypassing protective mechanisms [13,14,15,16]. This anatomical vulnerability becomes particularly important in urban environments where FA concentrations in air frequently exceed safe threshold values.

RAGE serves as a key molecular link between environmental exposure and neuroinflammatory pathology. Upon ligand binding, RAGE activates pro-inflammatory signaling cascades via nuclear factor kappa-B (NF-κB), mitogen-activated protein kinases (MAPK), and generation of reactive oxygen species (ROS), thereby forming a self-sustaining cycle of chronic neuroinflammation [17]. It is important to note that RAGE expression increases in response to oxidative stress and metabolic disturbances, creating a positive feedback loop that amplifies inflammatory responses in vulnerable brain regions such as the hippocampus [18].

In the context of AD pathogenesis, RAGE-mediated inflammation interacts with amyloidogenic processes through multiple mechanisms. First, RAGE facilitates the transport of Aβ across the BBB, increasing parenchymal Aβ burden [19,20,21]. Second, the interaction between RAGE and Aβ induces oxidative stress, which enhances the expression of BACE1, thereby shifting APP processing toward the amyloidogenic pathway [22]. Third, chronic activation of RAGE disrupts insulin signaling in the brain, contributing to the development of central insulin resistance, a condition increasingly recognized as an important factor in AD pathogenesis [23,24].

Previously, adverse effects associated with the accumulation of endogenous FA have been investigated. A relationship between FA exposure and AD development has been established, emphasizing the need to search for new diagnostic and therapeutic approaches [25]. It has been shown that degradation of FA reduces Aβ aggregation and improves cognitive function [26]. FA is released during memory formation from mitochondrial sarcosine dehydrogenase (SARDH). Subsequently, FA enhances current flow through N-methyl-D-aspartate receptors (NMDAR). However, at excessive concentrations, FA crosslinks NR1 and NR2B subunits of NMDAR [27]. Long-term exposure to FA negatively affects cognitive performance [28,29,30,31,32]. Most available data were derived from studies involving prolonged FA exposure (six weeks). Moreover, while an association between FA and hyperglycemia was reported, the biochemical mechanism responsible for this effect was not elucidated [28]. In contrast, industrial emissions and urban air pollution typically occur as short-term spikes rather than continuous exposure, while the neurological consequences of such acute or subacute FA exposure remain insufficiently studied. The role of FA in promoting amyloid plaque accumulation, ROS generation, and other neurodegenerative changes within a 7-day period has been demonstrated [31]. While this aligns with short-term FA exposure and may hold relevance for early diagnosis, the underlying mechanism driving Aβ accumulation was not identified in that study.

Recent studies have revealed a direct molecular link between FA exposure and RAGE activation. It has been demonstrated that FA modifies lysine residues in proteins, forming a specific adduct, identified in the hippocampus of mice following inhalation exposure, which acts as a ligand for RAGE [33]. Previously, a positive reaction between FA and peptides was recorded at 37 °C after 10–20 min of incubation [34]. In addition, the high reactivity of FA with insulin leading to adduct formation has been reported [35].

In the present study, we investigated relatively short-term FA exposure and developed a novel intranasal FA administration model that reproduces realistic environmental exposure scenarios in industrial cities. Although FA-induced upregulation of RAGE expression resulting from the formation of specific protein adducts has been previously described, our study focused on the insulin resistance component of this mechanism [33]. We have established a link between the toxic effects of FA and the insulin-dependent signaling pathway, providing a comprehensive description of both the mechanism underlying hyperglycemia and the direct impairment of neuroplasticity resulting from insulin resistance. We examined the biochemical mechanism of toxic effects of exogenous FA in the hippocampus leading to the development of insulin resistance due to the formation of FA-insulin adducts in the brain. We hypothesized that short-term (7-day) intranasal administration of FA, mimicking episodic air pollution exposure, would trigger RAGE-dependent neuroinflammatory cascades. In particular, we focused on the amyloidogenic branch of the RAGE signaling pathway, investigating how FA-induced insulin adduct formation and subsequent metabolic dysregulation lead to RAGE overexpression, alter the ADAM10/BACE1 balance, and promote amyloid deposition in the hippocampus. Previously, based on the literature data, we proposed a possible link between RAGE-mediated Aβ elevation associated with FA exposure and the development of insulin resistance [36]. Furthermore, the molecular mechanisms identified in this study support and extend the concept of type 3 diabetes [2,3]. By establishing a direct mechanistic connection between environmental FA exposure and AD-related pathology through the “lung-brain” axis, our findings provide new insight into how air pollutants may accelerate neurodegenerative processes in vulnerable populations. The mechanism we identified can develop within a short timeframe, which is particularly important for early diagnosis of pathological states in individuals susceptible to the toxic effects of FA.

## 2. Materials and Methods

### 2.1. Animals

All procedures for animal care and treatment were conducted in accordance with the regulations of Krasnoyarsk State Medical University and GOST 33215-2014 and were approved by the local ethics committee. All efforts were made to minimize animal suffering and to reduce the number of animals used in this study.

Two-month-old male CD-1 IGS WT mice (Charles River Laboratories, Wilmington, MA, USA) were used in this study. Animals were maintained under a 12 h light/dark cycle with free access to food and water.

### 2.2. Intranasal Administration

Prior to intranasal administration, mice were acclimated to prevent the development of anxiety-like behavior. During the procedure, the mouse was placed on the top of the cage. Using the dominant hand, the lower body was gently restrained by holding the tail against the cage surface, allowing the animal to grasp the bars. With the non-dominant hand, the skin across the shoulder blades was firmly grasped, avoiding excessive muscle stretching and pressure on the thorax. Using the index finger, the skin over the head was gently pulled backward to adjust head positioning. The mouse was then turned onto its back while stabilizing the tail. It was important to ensure that the neck and chin were parallel to the floor to facilitate intranasal delivery. Respiration was monitored during the procedure, and administration was paused if necessary [31,37].

The solution was administered in a volume of 10 μL daily for 7 days. The FA concentration corresponded to 6.5 MPC_one-time_ recorded in a large industrial center of the Russian Federation (Krasnoyarsk) in 2022. Considering that 1 MPC for FA equals 0.05 mg/m^3^, the calculated concentration was 0.325 mg/m^3^ (0.05 mg/m^3^ × 6.5) [38]. In 2-month-old CD-1 mice, the average minute ventilation is approximately 1.46 mL/g/min [39]. Based on this value, a mouse weighing 35 g has a minute ventilation of 51.1 mL/min, corresponding to a daily inhaled volume of 0.07 m^3^/day (73.5 L/day). At an atmospheric FA concentration of 0.325 mg/m^3^, the estimated daily inhaled dose is 0.02 mg per mouse (0.325 mg/m^3^ × 0.07 m^3^/day). The FA concentration = 0.02 mg/day (solution concentration: 2 mg/mL) corresponds to the FA1x group. To achieve a more pronounced effect, the concentration was increased tenfold to 0.2 mg/day (solution concentration: 20 mg/mL)—FA10x group.

To confirm the hypothesis of FA-insulin adduct formation, an additional FA10x + ins group was included, in which, in addition to the 10-fold FA solution, insulin (0.25 IU/kg) was administered intraperitoneally. The dose of 0.25 IU/kg is widely recognized as the lower bound of the standard insulin range for murine studies [40]. The control group received phosphate-buffered saline (PBS).

### 2.3. Blood Glucose Measurement

Blood glucose levels were measured using a glucometer (One Touch Select Flex, LifeScan, Zug, Switzerland) and corresponding test strips. Measurements were performed throughout the 7 days of intranasal administration. A small incision was made at the tip of the tail using a sterile lancet. Blood glucose levels were measured before and 15 min after intranasal administration. Animals were food-deprived for 4 h prior to the procedure.

### 2.4. Glucose Tolerance Test

The glucose tolerance test was performed on day 8 of the experiment (24 h after the last intranasal administration). A glucose solution (2 mg of glucose per gram of body weight) was used. The injection was performed at a 30–45° angle to avoid subcutaneous administration. Injections were made close to the midline to prevent damage to internal organs. Blood glucose levels were measured using the same glucometer before glucose administration and at 15, 30, 60, 90, and 120 min thereafter [41].

### 2.5. Colorimetric Detection of FA-Induced Protein Changes

This method is based on the Bradford assay, which allows for the detection of protein modifications [42,43]. An insulin solution at a concentration of 1.75 μg/mL was incubated with PBS, FA1x (0.02 mg), or FA10x (0.2 mg) for 15 min at 37 °C. FA-induced modifications of insulin were assessed using a colorimetric method. A protein assay kit (Bio-Rad, Hercules, CA, USA) was added to the samples, and optical density was measured at 595 nm using a multimode plate reader (CLARIOstar Plus, BMG LABTECH GmbH, Ortenberg, Germany). Insulin concentration was calculated based on optical density relative to a standard sample with a known concentration. A concentration-dependent decrease in detectable protein was interpreted as an indicator of FA-induced physicochemical modification of insulin

### 2.6. Behavioral Testing

#### 2.6.1. Passive Avoidance Test

Memory retention was assessed using the PA test. The apparatus consisted of a square acrylic chamber (18 × 18 × 26 cm) placed inside a sound-attenuating box (170 × 210 × 200 cm) to minimize external noise. The chamber was illuminated by LED lights (100 l×).

The testing protocol consisted of two days. On day 1, an association was formed between stepping down from an elevated platform (2 × 8 × 2 cm) and an aversive stimulus (foot shock). The animal was placed on the elevated platform, and when it stepped down, an electric shock was delivered. On day 2, the animal was placed in the same chamber without shock delivery. The time spent off the platform and the latency to the first step-down were recorded [44]. During testing, movement was automatically recorded using the ANY MAZE animal video analysis system.

#### 2.6.2. Radial Arm Maze Test

Working and long-term spatial memory were assessed using an 8-arm radial maze made of gray PVC. Each arm was 35 cm long and 6 cm wide. The central platform had a diameter of 20 cm. The walls were 15 cm high, and the maze was elevated 50 cm above the floor. Each arm was separated from the center by a guillotine door; a food reward was placed at the end of each arm, also equipped with a guillotine door. Animal behavior was recorded by a video camera mounted above the maze.

The protocol was described previously [45,46]. Briefly, it consisted of three stages:

Stage 1—the mouse was placed in the center with all arms open and baited with sugar, allowing free exploration for 5 min. This procedure was repeated three times with 30 s intervals.

Stage 2—four randomly selected arms were opened and baited. The mouse explored for 5 min. During the delay phase, the mouse remained in the center for 30 s. During testing, all arms were opened, but food was available only in previously closed arms. Exploration lasted 5 min.

Stage 3—repeat stage 2 after 60 min.

Stage 1 was performed only on the first testing day. Stages 2 and 3 were performed on days 1, 2, 3, 4, and 6. During testing, movement was recorded using the ANY MAZE system.

Average memory score depended on the number of correct and incorrect entries during testing. Correct entry (OK) was defined as a single entry into an arm. Incorrect entry (ERR) was defined as re-entry into the same arm. The average memory score was calculated as: Average memory score = (OK − ERR)/(OK + ERR).

#### 2.6.3. Elevated Plus Maze Test

The Elevated Plus Maze (EPM) was used to assess anxiety-like behavior as previously described [47,48]. The maze consisted of two open arms (6 cm × 32 cm) and two closed arms (6 cm × 32 cm with 19 cm high opaque walls) with a central area of 6 cm × 6 cm. The maze was elevated 54 cm above the floor. The room was dimly lit, and overhead lighting was used. The mouse was placed in the center facing an open arm and allowed to explore for 5 min. Time spent in closed arms was recorded. The maze was cleaned with 70% ethanol between trials. During testing, movement was automatically recorded using the ANY MAZE animal video analysis system.

### 2.7. Acute Slice Preparation

Mice were deeply anesthetized with Zoletil (50 mg/kg, Virbac, France) intraperitoneally and decapitated. The brain was rapidly removed and placed in ice-cold Ringer’s solution containing (in mM): 234 sucrose, 26 NaHCO_3_, 2.5 KCl, 1.25 NaH_2_PO_4_, 11 glucose, 10 MgSO_4_, and 0.5 CaCl_2_; pH 7.4, continuously oxygenated with 95% O_2_ and 5% CO_2_.

Hippocampal slices (350 μm thick) were prepared using a microslicer (Campden Instruments, Loughborough, Leicestershire, UK). Slices were maintained in extracellular solution containing (in mM): 125 NaCl, 2.5 KCl, 2 CaCl_2_, 1 MgCl_2_, 1.25 NaH_2_PO_4_, 26 NaHCO_3_, and 10 D-glucose. The solution was continuously oxygenated (95% O_2_/5% CO_2_) at room temperature for 1 h before electrophysiological recordings [49].

### 2.8. Field Excitatory Postsynaptic Potential Recording

To assess short-term synaptic plasticity, stimulation was applied to the CA1 region of the hippocampus at a frequency of 70 mHz. Field excitatory postsynaptic potentials (fEPSP) were recorded using electrodes filled with artificial cerebrospinal fluid. For paired-pulse facilitation (PPF) measurements, two electrical pulses (0.1 ms, 0.033 Hz) were delivered with a 50 ms interval. Electrophysiological data were analyzed using pClamp10 (Molecular Devices, San Jose, CA, USA), Patchmaster (HEKA), and Clampfit 10.5 (Axon Instruments, Union City, CA, USA) [50].

### 2.9. Mouse Tissue Processing

Histological sample preparation was performed using the manual tissue processing method. Fixation was carried out using 10% neutral buffered formalin. Tissue samples were immersed in the fixative solution for 24 h at room temperature. Subsequently, the samples were washed in running tap water for 1 h. Tissue processing (dehydration) was performed using isopropyl alcohol in ascending concentrations (50%, 70%, 96%, 100%, 100%), with samples remaining in each solution for 2 to 4 h. Paraffin (Merck & Co., Inc., Rahway, NJ, USA) was used as the embedding medium. Infiltration involved three changes of paraffin (for 1, 3, and 4 h, respectively) in a thermostat at 60 °C. Finally, the samples were embedded in paraffin using wooden molds. Before slicing, the brain was heated in a water bath at 40 °C. Thin sections of 20 µm were obtained using a rotary microtome (Leica Biosystems Nussloch GmbH, Nussloch, Germany). Deparaffinization was performed by immersing the sections in xylene (three changes, 10 min each), followed by rehydration in a descending ethanol series (96%, 70%, 50%; 3 min in each). Afterwards, the sections were rinsed in two changes of distilled water (3 min each).

### 2.10. Immunohistochemistry (IHC)

For IHC, mice were terminally anesthetized with Zoletil and perfused transcardially with a fixative containing 4% paraformaldehyde in 0.1 M phosphate buffer. The whole brain was removed and postfixed in the same fixative for 5–6 h or overnight. For IHC on anti-c-Fos, the brain was fixed 90 min after training in the PA test (1 day). The hippocampus was cut into 50 µm coronal slices (Campden Loughborough, UK). The sections were treated with rabbit polyclonal anti-RAGE (ab3611), anti-c-Fos (ab190289), (1:500, Abcam, Cambridge, UK), mice polyclonal anti-NeuN (1:500, Abcam, EPR12763, Cambridge, UK). Secondary antibodies Alexa Fluor 555-conjugated goat anti-rabbit IgG (1:500, Abcam, ab150086, Cambridge, UK), or Alexa Fluor 488-conjugated goat anti-rabbit IgG (1:500, Abcam, ab150077, Cambridge, UK) were used. The antibodies were dissolved in a PBS solution containing 2% (*v/v*) normal donkey serum, 0.1% (*v/v*) Triton X- 100, and 0.05% NaN3. For mounting under a coverslip, a 50% solution of non-fluorescing glycerol in phosphate buffer containing DAPI was used as the mounting medium.

### 2.11. Fluorescent Staining

To detect amyloid deposits with high sensitivity and specificity, we employed an optimized Th-S staining protocol [51]. This protocol is optimized for detecting amyloid deposits at an early stage. Briefly, Th-S was dissolved in distilled water to prepare a filtered 1% stock solution, which was stored at –20 °C protected from light. Working solution was freshly prepared by diluting the stock in phosphate-buffered saline (PBS) to 1 × 10^−5^% (g/100 mL) concentration. The slices were stained for 24 h at room temperature and protected from light. Then they were rinsed in PBS and mounted in 50% PBS:glycerol.

### 2.12. Microscopy

In all groups, the hippocampal areas CA1, CA3 and DG were used for comparisons. Fluorescent images were obtained using FV10i Confocal Microscope (Olympus, Tokyo, Japan) with a resolution (10× objective, 4× magnification). Images from the same confocal plane and under the same exposure and gain were compared to assess double labeling. A fluorescent microscope was used to obtain images of amyloid deposits (ZOE, Fluorescent Cell Imager, Bio-Rad Laboratories, Hercules, CA, USA) (480 nm).

### 2.13. Real-Time Polymerase Chain Reaction

For real-time PCR, the hippocampus was isolated separately. The tissue was stored in an RNA stabilizer (Biolabmix, Novosibirsk, RU) at −80 °C. Next, RNA was isolated using a ready-made kit for isolating total RNA and microRNA from the Lira reagent on columns (Biolabmix, Novosibirsk, RU) according to the manufacturer’s instructions. The concentration and purity of the obtained RNA were measured using a spectrophotometer (NanoVue Plus, GE Healthcare, Little Chalfont, UK). Reverse transcription was performed using 7 μL of total RNA and the ready-to-use M-MuLV-RH kit (Biolabmix, Novosibirsk, RU) according to the manufacturer’s instructions. Real-time PCR was performed using the ready-to-use 5X qPCRmix-HS SYBR PCR mixture (Eurogen, Moscow, RU) and the LightCycler 96 amplifier (Roche, Basel, CH). The genes studied were ADAM10: forward 5′-TCATGGTGAAACGCATAAGAATC-3′, reverse 5′-CAACTCCAGGAACTTCTCCACA-3′ and BACE1: forward 5′-GCCTATGCTGAGATTGCCAG-3′, reverse 5′-GCAGGGAAAAGATGTTGGGA-3′ (DNA-Synthesis, Moscow, RU). The expression levels of the studied genes were calculated relative to the expression of the reference genes GAPDH, ACTB (Primer sequences are commercially confidential (DNA-Synthesis, Moscow, RU)): using the 2^−ΔΔCt^ method.

### 2.14. Analysis of Aß Monomer Levels

To analyze the level of Aβ monomers, we used the Mouse Aβ42 ELISA Kit, Fine test, following the manufacturer’s instructions. Briefly, the hippocampal tissue was homogenized in 500 μL of PBS. 100 μL of the standard or sample was used. After incubation for 90 min at 37 °C, the plate was washed twice without immersion. Then, 100 μL of the biotinylated antibody working solution was added to each well containing the standard or sample. After incubation for 60 min at 37 °C, the plate was washed three times for 1 min each. Then, 100 μL of the SABC working solution was added, the plate was covered, and incubated for 30 min at 37 °C, followed by five washes for 1 min each. Finally, 90 μL of the TMB substrate solution was added, the plate was covered, and incubated for 10–20 min at 37 °C. Finally, 50 μL of stop solution was added, and the optical density was measured at 450 nm (CLARIOstar Plus, BMG LABTECH GmbH, Ortenberg, Germany) for subsequent calculation of the results.

### 2.15. Statistical Analysis

Data following a normal distribution are presented as mean ± standard error of the mean (SEM). Data that did not meet the assumptions of normality are presented as median with the corresponding interquartile range (IQR). Statistical analyses were performed using GraphPad Prism (version 10.6.0., GraphPad Software, San Diego, CA, USA) and/or SPSS (version 31.0, IBM, Armonk, NY, USA). The threshold for statistical significance was set at *p* < 0.05. Normality of data distribution was assessed using the Shapiro–Wilk test. Homogeneity of variances was evaluated using Levene’s test. When assumptions of normality and homoscedasticity were met, parametric tests were applied. In cases where these assumptions were violated, appropriate non-parametric alternatives were used.

For comparisons between more than two independent groups (PBS, FA1× FA10×, FA10× + ins), one-way analysis of variance (ANOVA) was used, followed by Tukey’s post hoc multiple comparisons test. When data were not normally distributed, the Kruskal–Wallis test was performed, followed by Dunn’s post hoc test with correction for multiple comparisons.

Repeated-measures data (e.g., glucose levels across multiple days or time points in glucose tolerance test; learning curves in RAM) were analyzed using two-way repeated-measures ANOVA, with treatment as the between-subject factor and time/day as the within-subject factor. When significant main effects or interactions were detected, post hoc comparisons were conducted using Tukey’s test with adjustment for multiple testing.

For paired comparisons within the same animals (e.g., glucose levels before and 15 min after intranasal administration), paired Student’s *t*-test was used for normally distributed data. If normality was not met, the Wilcoxon matched-pairs signed-rank test was applied.

Electrophysiological parameters (PPF ratios) and gene expression data (ΔΔCt values) were analyzed using one-way ANOVA or Kruskal–Wallis test, as appropriate.

Outliers were identified using the ROUT method (Q = 1%) and excluded only if statistically justified. All exclusions were reported.

Sample sizes (n) refer to the number of animals per group unless otherwise specified. No statistical methods were used to predetermine sample size; however, group sizes were based on previous studies using similar behavioral and molecular endpoints.

## 3. Results

### 3.1. FA Exposure Impairs Memory Performance in CD1 Mice

**Figure 1:**
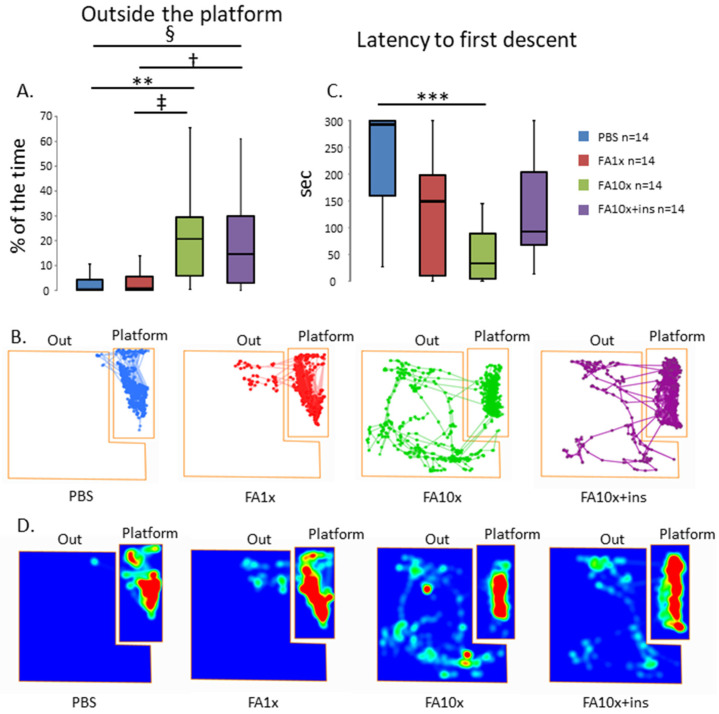
FA exposure induces cognitive deficits in the PA test. (**A**) FA10x group spent more time outside the platform (** *p* = 0.007, † *p* = 0.02, ‡ *p* = 0.01, § *p* = 0.01, Kruskal–Wallis post hoc Dunn’s test), *n*—number of examined animals. (**B**) Representative travel pathways show the position of the animal’s center point for the entire duration of the test. (**C**) FA10x spends less time on the platform before the first descent (*** *p* = 0.0005 Kruskal–Wallis post hoc Dunn’s test), *n*—number of examined animals. (**D**) Representative heat maps show the duration of stay in the test zones.

To investigate the effects of FA on cognitive processes, we used the PA test. Mice exposed to the 10-fold FA concentration spent more time off the elevated platform (% time off platform) (Figure 1A,B). In addition, with increasing FA concentration, the latency to first step-down from the platform (seconds) decreased (Figure 1C,D). In the FA10x + ins group, the time spent on the elevated platform increased slightly. However, the time spent off the platform in these animals did not differ from the FA10x group without insulin administration.

**Figure 2:**
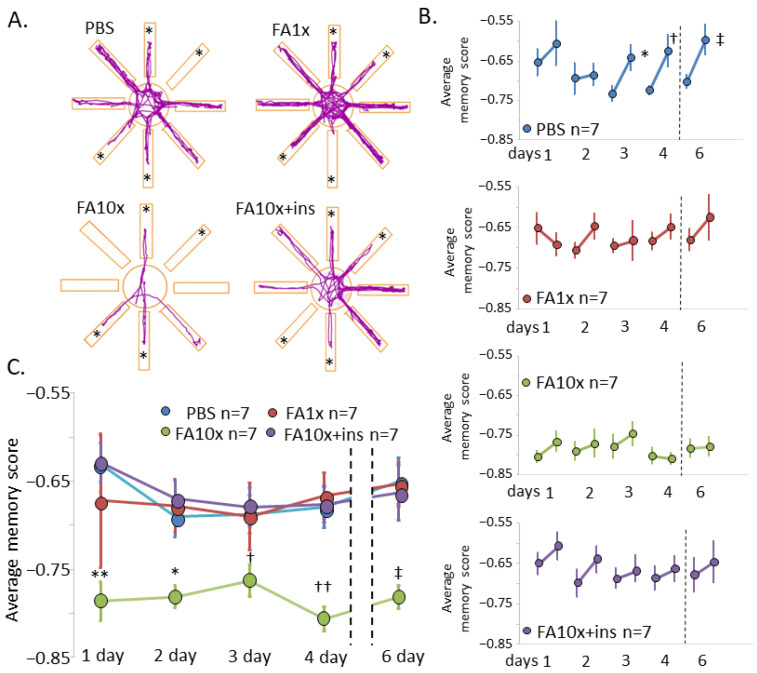
Both low and high doses of FA impair spatial working memory in the RAM. (**A**) Representative travel pathways show the position of the animal’s center point for the entire duration of the test (day 6). *—open arms at the training stage. (**B**) Working memory results. Each point represents the average memory score of the 2nd and 3rd stages of the test in the testing phase on a single day. The results showed no significant difference in the FA1x, FA10x, and FA10x + ins groups in terms of the average memory score between stages 2 and 3 on all testing days (* *p* = 0.03, † *p* = 0.05, ‡ *p* = 0.04, paired samples *t*-test). *n*—number of examined animals. (**C**) Long-term memory results. Each point represents the average memory score in the testing phase for one day, which depends on the number of correct and incorrect entries into the arms. The results showed a decrease in spatial memory in the FA10x group starting from the first day of testing. Adding insulin allowed for the restoration of memory efficiency (** *p* = 0.002, * *p* = 0.03, † *p* = 0.05, †† *p* = 0.001, ‡ *p* = 0.002, Kruskal–Wallis post hoc Dunn’s test). *n*—number of examined animals.

For a more detailed analysis of working memory, we examined mouse behavior in the RAM (Figure 2A). Control mice demonstrated effective learning and located the food reward significantly better on training days 3, 4, and 6. Working memory was impaired in both FA1x and FA10x groups and was not improved by insulin administration (FA10x + ins group) (Figure 2B). Long-term memory was impaired only in the FA10x group across all training days. Insulin administration restored this parameter to control levels (Figure 2C).

To confirm the absence of anxiety induced by the intranasal administration procedure, the EPM test was performed, including an additional naïve control group (animals from home cages). No anxiety-like behavior was observed in any of the experimental groups (Supplementary Figure S1).

### 3.2. FA Exposure Reduces Hippocampal Neuronal Activity During Learning

**Figure 3:**
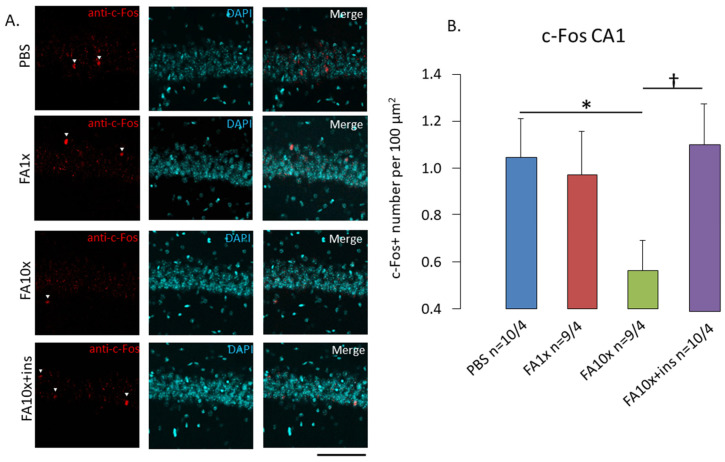
FA10x exposure reduces c-Fos expression in CA1 hippocampal neurons. (**A**) Representative images c-fos (red), DAPI (blue) and colocation c-Fos-DAPI (merge) in CA1 neurons. Scale bar 100 μm. White triangles indicate c-Fos-positive cells that were included in the analysis. (**B**) The graph shows mean ± SEM of c-Fos-positive cells per 100 µm^2^. *n*—number of examined slices/animals. * *p* = 0.02, † *p* = 0.01, ANOVA method and the Tukey post hoc test.

To assess hippocampal neuronal activity during learning, we quantified the number of c-Fos-positive cells in the pyramidal layer of the CA1, CA3, and dentate gyrus (DG) regions. A significant reduction in c-Fos-positive cells was observed in all hippocampal regions in the FA10x group (Figure 3, Supplementary Figures S2 and S3). Insulin administration increased the number of c-Fos-positive cells in the CA1 region to control levels (Figure 3).

### 3.3. Intranasal FA Administration Reduces Short-Term Synaptic Plasticity in Mice

**Figure 4:**
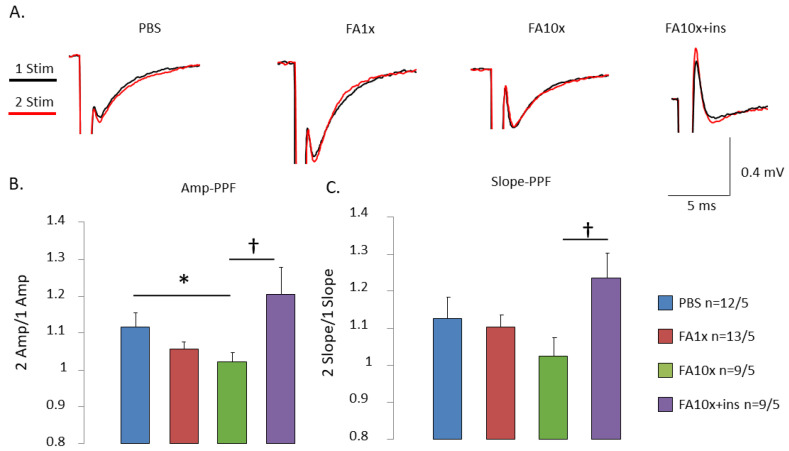
Intranasal FA administration impairs short-term synaptic plasticity in CA1 hippocampal neurons. The results of the average PPF values. (**A**) Representative fEPSC curves, 1 stimulus (black) and 2 stimulus (red). (**B**) The graph shows mean ± SEM of PPF ratio (1st Amp/2nd Amp) after intranasal injection of FA. The decrease in the FA10x group is significant. Adding insulin (FA10x + ins) increased the PPF value (* *p* = 0.05, † *p* = 0.04, ANOVA method and the Tukey post hoc test), *n*—number of examined slices/animals. (**C**) The graph shows mean ± SEM of PPF ratio (2nd Slope/1st Slope). Only an increase in slope-PPF in the FA10x + ins group compared to the FA10x group is significant († *p* = 0.05, ANOVA method and the Tukey post hoc test), *n*—number of examined slices/animals.

**Figure 5:**
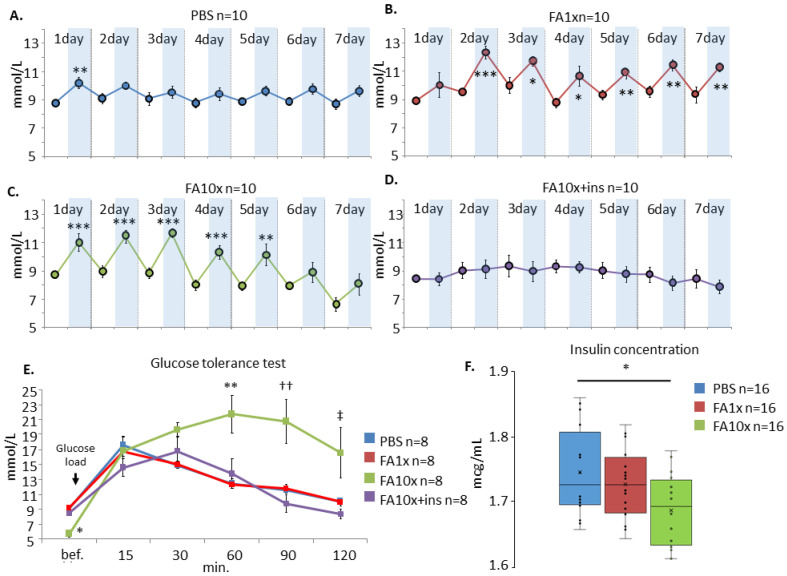
Intranasal FA induces acute hyperglycemia and persistent glucose intolerance. (**A**) On the first day, the PBS group showed a statistically significant increase in glucose levels after 15 min, due to the stress response to the intranasal injection procedure itself (paired samples *t*-test). *n*—number of examined animals. ** *p* = 0.006, ANOVA method and the Tukey post hoc test. White line—before intranasal administration. Blue line—15 min after intranasal administration. (**B**) There was a significant increase in glucose concentration in the FA1x after intranasal administration of the solution (day 2–7). *n*—number of examined animals. * *p* < 0.05, ** *p* < 0.01, *** *p* < 0.001. White line—before intranasal administration. Blue line—15 min after intranasal administration. (**C**) The same effect was observed in the FA10x from day 1 to day 5, but not on days 6 and 7 (paired samples t-test). *n*—number of examined animals. ** *p* < 0.01, *** *p* < 0.001. White line—before intranasal administration. Blue line—15 min after intranasal administration. (**D**) The administration of insulin made it possible to neutralize this negative effect (FA10x + ins). n—number of examined animals. White line—before intranasal administration. Blue line—15 min after intranasal administration. (**E**) Glucose tolerance test. A significant violation of glucose metabolism in FA10x was revealed. * *p* = 0.03, ** *p* = 0.001, †† *p* = 0.006, ‡ *p* = 0.02, Kruskal–Wallis post hoc Dunn’s test. *n*—number of examined animals. (**F**) Measurement of insulin concentration using colorimetric analysis after incubation (15 min.) with FA. A decrease in concentration is shown in response to an increase in FA concentration, indicating FA-induced modification of the insulin. Each point shows the concentration of the sample. * *p* = 0.02, ANOVA method and the Tukey post hoc test. *n*—number of examined samples.

Short-term synaptic plasticity was evaluated using PPF in hippocampal slices obtained after 7 days of intranasal FA administration. The PPF ratio calculated by amplitude (amp-PPF ratio) was significantly decreased in hippocampal slices from FA10x mice (Figure 4A,B). However, the PPF ratio calculated by slope (slope-PPF ratio) did not change significantly in these animals (Figure 5A,C). Insulin administration significantly affected synaptic plasticity, increasing both amp-PPF and slope-PPF ratios (Figure 4A–C).

### 3.4. FA-Induced Hyperglycemia

It is known that FA has the chemical reactivity to form adducts with insulin [35]. We investigated the dynamics of blood glucose concentration under normal conditions and in response to acute FA exposure. On the first day, the PBS group showed a statistically significant increase in glucose levels after 15 min, likely due to the stress response associated with the intranasal administration procedure itself. However, on subsequent days, such fluctuations in glucose levels were not observed (Figure 5A).

A significant increase in blood glucose levels 15 min after intranasal administration was observed in the FA1x group from days 2 to 7 (Figure 5B), as well as in the FA10x group from days 1 to 5 (Figure 5C). In the FA10x group, glucose levels began to decrease starting from day 4 of the experiment (Figure 5C). In contrast, no statistically significant increases in glucose levels were detected in the FA10x + ins group (Figure 5D).

After completion of the intranasal administration protocol, a glucose tolerance test was performed the following day. In the FA10x group, a sustained elevation of blood glucose levels after glucose loading was observed for at least 2 h (Figure 5E).

To determine the cause of the observed impairment in glucose metabolism, we assessed the level of free insulin in the presence of FA, which is capable of forming adducts with proteins, including insulin. A concentration-dependent modification of insulin was observed after 15 min of incubation at 37 °C (Figure 5F).

### 3.5. Impaired Glucose Metabolism Increases RAGE Expression in the Hippocampus

**Figure 6:**
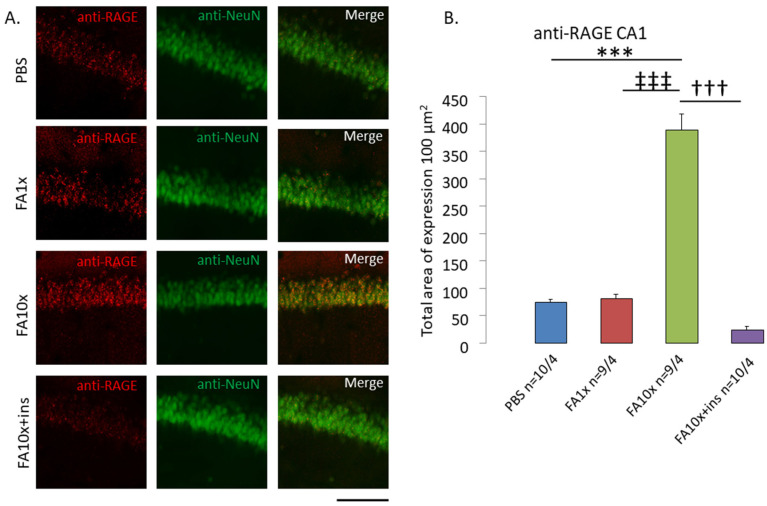
Intranasal FA increases RAGE-positive area in CA1 hippocampal region. (**A**) Representative images of RAGE (red), NeuN (greed), colocation RAGE-NeuN (merge) in CA1 neurons. Scale bar 100 μm. (**B**) The results show an increase in RAGE expression in the FA10x group, and a decrease when insulin is added. *n*—number of examined slices/animals. *** *p* < 0.001, ††† *p* < 0.001, ‡‡‡ *p* < 0.001, ANOVA method and the Tukey post hoc test.

Prolonged elevation of blood glucose levels leads to increased formation of advanced glycation end products (AGEs) and RAGE located on neuronal membranes. In this regard, we examined the area of RAGE expression in the hippocampus (Figure 6A).

A significant increase in the RAGE-positive area was detected in the CA1 region in FA10x animals. Insulin administration markedly reduced RAGE expression (Figure 6B). In other hippocampal regions, CA3 and DG, an increase in RAGE expression was also observed in the FA10x group (Supplementary Figures S4 and S5). These findings demonstrate that the RAGE-positive area extended across all hippocampal regions in FA10x animals.

Upon insulin administration and reduction in blood glucose levels, the level of glycated proteins decreased, resulting in a reduced number of cells expressing activated RAGE on their surface.

### 3.6. FA Accumulation Disrupts APP Processing in the Hippocampus and Promotes Amyloid Deposition

RAGE directly influences APP processing through modulation of β- and γ-secretase activity [52]. ADAM10 and BACE1 are APP-processing genes responsible for the synthesis of α-secretase and β-secretase, respectively. Their ratio is critical for maintaining normal levels of Aβ42 monomers. Disruption of this balance leads to Aβ accumulation, which is associated with amyloid deposition.

**Figure 7:**
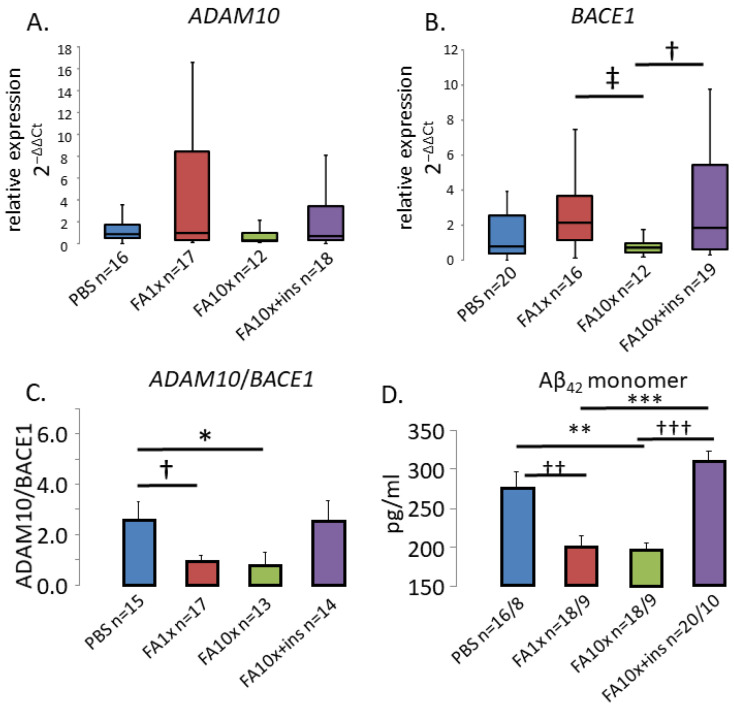
FA exposure alters ADAM10/BACE1 expression balance and reduces Aβ42 monomer levels in the hippocampus. (**A**) The expression of the ADAM10 gene was increased in the FA1x group. *n*—number of examined samples. (**B**) The expression of the ADAM10 gene was increased in the FA1x and FA10x + ins groups. ‡ *p* = 0.04, † *p* = 0.04, Kruskal–Wallis post hoc Dunn’s test. (**C**) The ratio of ADAM10/BACE1 gene expression. The ratio of genes in the FA1x and FA10x groups was shown to decrease. *n*—number of examined slices. * *p* = 0.04, † *p* = 0.05, ANOVA method and the Tukey post hoc test. (**D**) Results of ELISA measurements of Aβ42 monomer. A decrease in Aβ42 monomer concentration is shown in the FA1x and FA10x groups. *n*—number of examined samples/animals. The addition of insulin increased the concentration of Aβ42 monomer. †† *p* = 0.006, ** *p* = 0.002, *** *p* < 0.001, ††† *p* < 0.001, ANOVA method and the Tukey post hoc test.

RT-PCR analysis of individual genes did not reveal changes in ADAM10 expression compared with PBS-treated control animals (Figure 7A). However, a statistically significant increase in BACE1 expression was observed in the FA1x and FA10x + ins groups relative to the FA10x, but not with the PBS (Figure 7B). At the same time, a significant decrease in the ADAM10/BACE1 expression ratio was observed in the FA-treated groups (FA1x and FA10x) (Figure 7C).

Increased BACE1 expression would be expected to lead to accumulation of Aβ42 monomers and subsequent oligomerization. However, in these animals, the level of Aβ42 monomers in the hippocampus was decreased (Figure 7D). Based on these findings, we hypothesized that synthesis of these monomers was not reduced; rather, they were undergoing oligomerization and forming amyloid deposits in brain structures.

**Figure 8:**
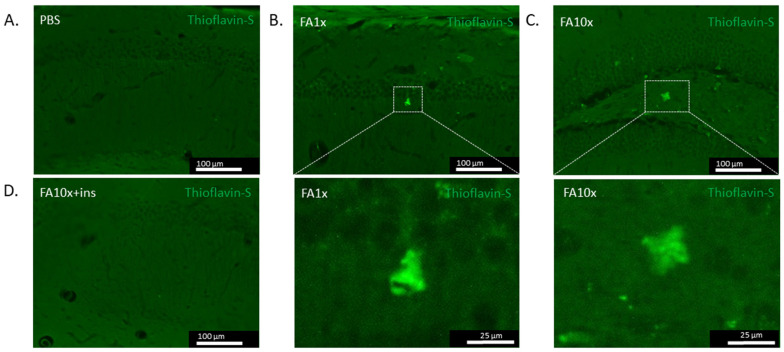
One-week FA exposure reduces Aβ42 monomers and promotes amyloid plaque formation. (**A**–**D**) Representative images of Th-S staining. (**A**,**D**) Absence of amyloid plaques in PBS and FA10x + ins group. (**B**,**C**) Amyloid plaques detected in FA1x and FA10x groups.

We therefore analyzed the presence of amyloid deposits in the hippocampus using Th-S histochemical staining. The results demonstrated the presence of amyloid deposits in the FA1x and FA10x groups (Figure 8). Thus, after 7 days of intranasal FA administration, a disruption in the ADAM10/BACE1 expression balance is formed, leading to decreased Aβ42 monomer levels due to their oligomerization and amyloid deposits formation. No amyloid deposits were detected in the hippocampus of FA10x + ins mice, which is consistent with our previous findings (Figure 8).

## 4. Discussion

Our study demonstrates that short-term intranasal exposure to FA, mimicking realistic environmental pollution scenarios in industrial cities, triggers a cascade of neuroinflammatory and metabolic disturbances culminating in amyloidogenic pathology and cognitive impairment. The key mechanistic insight lies in the finding that FA forms adducts with insulin, leading to acute hyperglycemia, sustained overexpression of RAGE, disruption of the ADAM10/BACE1 balance, and subsequent amyloid deposits formation in the hippocampus. Importantly, we showed that even a single-level concentration of FA (FA1x), corresponding to actual atmospheric levels recorded in a large industrial city of the Russian Federation (Krasnoyarsk) in 2022 [38], is sufficient to induce measurable impairments in working memory.

Previous studies have demonstrated that prolonged (6-week) exposure to FA negatively affects cognitive performance in mice [28]. In contrast, the duration of exposure used in the present study reflects relatively short-term fluctuations of FA concentration in the air of large industrial cities. Industrial FA emissions are typically intermittent and transient. In this study, we considered both the duration and intensity of FA pollution in a major industrial center of the Russian Federation (Krasnoyarsk) and evaluated its effects on memory performance in mice after 7 days of intranasal administration. For this purpose, the PA test and RAM were employed.

The behavioral consequences of FA exposure were dose-dependent, yet functionally significant at both tested concentrations. Mice exposed to the tenfold concentration (FA10x) exhibited pronounced impairments in both the PA and RAM performance, consistent with hippocampal dysfunction. These animals showed a higher percentage of time spent off the platform and reduced latency to first descent. Additionally, no improvement in average memory score was observed in the FA10x and FA10x + ins groups between test stages throughout the experimental period.

A similar pattern was observed in the FA1x group, which represents one of the most striking findings, as these mice were exposed to an environmentally relevant concentration. Although these animals demonstrated relatively preserved long-term memory performance, they failed to improve memory indices between the second and third testing stages across all experimental days. This pattern suggests a selective deficit in working memory, short-term information retention, and temporal encoding, rather than a generalized inability to learn.

At the same time, the FA10x group displayed more pronounced impairments across all test parameters. Interestingly, the addition of insulin (FA10x + ins) resulted in significant cognitive improvement only in the RAM test. However, the latency to first descent, the percentage of time spent off the platform, and the difference in average memory score between stages 2 and 3 remained unchanged.

Immunohistochemical analysis demonstrated a reduction in c-Fos expression in neurons of the CA1, CA3, and dentate gyrus (DG) regions of the hippocampus in the FA1x and FA10x groups during the formation of an association between spatial context (stepping down from the platform) and the aversive stimulus (foot shock), indicating reduced neuronal activity. According to the literature, such a decrease may be associated with insulin deficiency, which reduces phosphorylation of insulin receptor substrates 1/2 (IRS1/2), activation of growth factor receptor-bound protein 2 (GRB2) and subsequent activation of the Ras-mediated pathway responsible for regulating the transcription factor cAMP response element-binding protein (CREB) and the expression of immediate early genes such as c-Myc, c-Jun, and c-Fos [36,53].

It should be noted that the relatively low c-Fos expression in CA1 is consistent with the binary nature of the PA test, which does not require complex spatial integration. Higher c-Fos levels in CA3 and DG reflect the involvement of these subregions in simple associative learning, in line with the functional specialization of the hippocampus [54,55].

A particularly compelling aspect of our results is the functional link between deficits in presynaptic plasticity and behavioral impairments. The decrease in PPF-amp in the FA10x group indicates impaired presynaptic short-term plasticity, likely due to disrupted calcium homeostasis and increased basal release probability. This interpretation is supported by the preservation of slope-dependent PPF, which remained unchanged, suggesting that the primary deficit lies in presynaptic vesicle dynamics rather than in postsynaptic receptor function. Restoration of both parameters following insulin administration further supports insulin deficiency is a driving factor. Notably, the shape of the response curve was altered, with a prolonged decay time, consistent with the known role of insulin in modulating GABAergic inhibition [56,57]. The correlation between electrophysiological and behavioral impairments supports our conclusion that FA-induced insulin disruption undermines synaptic mechanisms underlying working memory.

Within 15 min of intranasal administration, significant hyperglycemia was observed in both FA1x and FA10x groups, indicating functional insulin deficiency. This metabolic disturbance led to pronounced RAGE overexpression throughout the hippocampus. Compensation with exogenous insulin (FA10x + ins) restored glucose metabolism and significantly reduced hippocampal RAGE expression. Consistent with these in vivo findings, FA-induced insulin modification was detected within 15 min in vitro.

Previous studies have also demonstrated that intranasal FA exposure leads to Aβ accumulation and amyloid plaque formation [28,31]. However, the detailed molecular mechanism leading to this pathological condition remains insufficiently understood. To more precisely analyze APP processing, we performed qPCR analysis of ADAM10 and BACE1 gene expression. We detected a significant increase in BACE1 mRNA expression in the FA1x group, whereas ADAM10 expression remained unchanged. In contrast, in the FA10x group, expression levels of both genes did not differ from controls. Hyperglycemia is known to be a key factor promoting ADAM10 and BACE1 gene expression [58,59], which may explain the increased BACE1 expression in the FA1x group. However, mice in the FA10x group exhibited hypoglycemia beginning on day 4 of the experiment, which may exert the opposite effect on the expression of these genes.

In the FA10x + ins group, increased BACE1 expression was observed. This effect appears to be indirect and compensatory. BACE1 is known to act as a negative regulator of the insulin receptor (IR) by promoting its cleavage [60]. We hypothesize that additional insulin administration may have induced compensatory upregulation of BACE1 expression in order to reduce excessive insulin signaling by further decreasing the number of IR molecules on the cell membrane.

ADAM10 and BACE1 compete in APP processing [13]. Although absolute mRNA levels of ADAM10 and BACE1 varied among groups, the most informative indicator is not their individual expression levels but the calculated ADAM10/BACE1 ratio. A critical finding of this study was the decrease in the ADAM10/BACE1 expression ratio in the FA1x and FA10x groups. These data demonstrate the predominance of BACE1 expression in FA-treated groups. Aβ is one of the ligands of RAGE [61]. Enhanced interaction between Aβ and RAGE increases the production of ROS. Oxidative stress promotes increased expression of APP and BACE1 [22,62], directly affecting the ADAM10/BACE1 balance. This mechanism represents one of the factors contributing to enhanced APP processing and elevated Aβ levels. In addition, RAGE mediates Aβ transport into brain tissue across BBB [19,21]. Therefore, the observed RAGE overexpression and predominance of BACE1 in the hippocampus represent substantial factors contributing to increased Aβ levels.

The predominance of BACE1 expression and RAGE overexpression would suggest a subsequent increase in Aβ levels. However, ELISA analysis revealed a decrease in Aβ monomer levels in the hippocampus of the FA1x and FA10x groups. This effect can be explained by the inability of the ELISA assay to detect aggregates, as it was designed for quantitative measurement of monomeric proteins. Following histochemical analysis of hippocampal sections stained with Th-S, amyloid deposits were detected in the FA-treated groups. Importantly, we employed a modified ThS staining protocol that is specifically designed to detect early, diffuse amyloid deposits [63]. This approach enabled us to visualize more subtle aggregates that precede mature amyloid plaque formation. These data also explain the observed reduction in Aβ monomer levels. RAGE-mediated disruption of the ADAM10/BACE1 expression balance within one week results in rapid Aβ accumulation, which appears sufficient for amyloid deposit formation.

Insulin possesses direct neuroprotective effects, including inhibition of apoptosis and autophagy, as well as reduction in neuroinflammation [64,65]. It is important to note that many of these neuroprotective effects require preconditioning. In our study, insulin was administered concurrently with FA, rather than as a pretreatment. Although direct neuroprotective actions of insulin cannot be entirely ruled out, the concurrent administration paradigm employed in our study serves to mitigate their potential contribution to the protective effects observed.

Several methodological advantages enhance the practical relevance of our model. First, the intranasal route directly reproduces the primary pathway by which airborne pollutants affect humans, bypassing the BBB via the olfactory pathway and delivering FA directly to brain parenchyma. Second, the use of environmentally realistic concentrations—particularly the FA1x group corresponding to actual atmospheric measurements—improves the ecological validity of our findings. Third, the short duration of exposure (7 days) models episodic pollution events rather than chronic continuous exposure, thereby better reflecting real-world scenarios in industrial cities where FA emissions occur in spikes. Finally, the comprehensive multi-level assessment—from molecular and electrophysiological to behavioral outcomes—provides an integrated understanding of the pathological cascade triggered by environmental FA exposure.

Despite these strengths, certain limitations must be acknowledged. The study specifically focused on the amyloidogenic branch of the RAGE signaling pathway, leaving other inflammatory pathways (such as NF-κB activation and cytokine production) unexplored. Future studies should investigate the broader neuroinflammatory response, including microglial activation and astrocyte reactivity. In addition, although our electrophysiological analysis identified deficits in presynaptic plasticity, the absence of long-term potentiation (LTP) measurements limits our understanding of postsynaptic adaptive mechanisms. However, this limitation is partially offset by our clear demonstration that impairment of short-term presynaptic plasticity is sufficient to explain the observed behavioral deficits, particularly in working memory tasks that strongly depend on rapid synaptic modulation.

## 5. Conclusions

In conclusion, our results establish a direct mechanistic link between environmental FA exposure and AD-related pathology through the “lung-brain” axis. The cascade begins with the formation of insulin adducts, progresses through RAGE-mediated neuroinflammation and amyloidogenic APP processing, and culminates in synaptic dysfunction and cognitive impairment. These findings provide experimental support for the type 3 diabetes concept, which posits that AD may arise from impaired insulin signaling in the brain. By demonstrating that FA-induced insulin modification triggers the same downstream pathological events, our study offers a mechanistic foundation linking environmental exposure to this emerging disease paradigm. The demonstration that even environmentally realistic FA concentrations can induce measurable working memory deficits within only one week of exposure has important implications for public health policy and environmental regulation. These findings underscore the urgent need for monitoring and controlling FA levels in industrial regions, particularly in light of accumulating evidence linking environmental pollutants to increased risk of neurodegenerative diseases. A promising direction for future research is the investigation of similar mechanisms in humans under chronic FA exposure, as well as the evaluation of early interventions targeting insulin or RAGE signaling to mitigate adverse outcomes.

## Data Availability

Data will be made available on request.

## Supplementary Materials

The following supporting information can be downloaded at: https://www.mdpi.com/article/10.3390/biomedicines14040779/s1, Figure S1: A. Intranasal administration procedure does not induce anxiety in the elevated plus-maze test. B. Representative graph shows the position of the animal’s centre point for the entire duration of the test. n—number of examined animals. Statistical analysis was performed using the Kruskal Wallis post-hoc Dunn’s test; Figure S2: FA10x exposure reduces c-Fos expression in CA3 hippocampal neurons; Figure S3: FA10x exposure reduces c-Fos expression in DG neurons; Figure S4: Intranasal formaldehyde increases RAGE expression in CA3 hippocampal neurons. Figure S5: Intranasal formaldehyde increases RAGE expression in dentate gyrus neurons.

## Abbreviations

The following abbreviations are used in this manuscript:

AD	Alzheimer’s Disease
FA	Formaldehyde
RAGE	Receptor for Advanced Glycation End Products
APP	Amyloid Precursor Protein
Aβ	Beta-Amyloid
BACE1	Beta-site APP-Cleaving Enzyme 1
ADAM10	A Disintegrin And Metalloproteinase domain-containing protein 10
AGEs	Advanced Glycation End Products
CNS	Central Nervous System
BBB	Blood–Brain Barrier
MPC	Maximum Permissible Concentration
PA	Passive Avoidance
RAM	Radial Arm Maze
EPM	Elevated Plus Maze
fEPSP	Field Excitatory Postsynaptic Potential
PPF	Paired-Pulse Facilitation
Th-S	Thioflavin-S
NF-κB	Nuclear Factor kappa-B
MAPK	Mitogen-Activated Protein Kinases
ROS	Reactive Oxygen Species
IR	Insulin Receptor
IRS1/2	Insulin Receptor Substrate 1/2
CREB	cAMP Response Element-Binding Protein
LTP	Long-Term Potentiation
NMDAR	N-methyl-D-aspartate receptors
SARDH	Sarcosine Dehydrogenase
GRB2	Growth Factor Receptor-Bound protein 2
