# Supplementary material for: Intranasal Formaldehyde Exposure Induces RAGE-Mediated Alteration of the ADAM10/BACE1 Expression Balance and Amyloid Deposition
Source: Biomedicines. 2026 Mar 30;14(4):779. doi: 10.3390/biomedicines14040779 (PMC13113879; doi:10.3390/biomedicines14040779)
Supplement: Supplementary file 1 [file biomedicines-14-00779-s001.zip › biomedicines-4212132-supplementary.pdf]

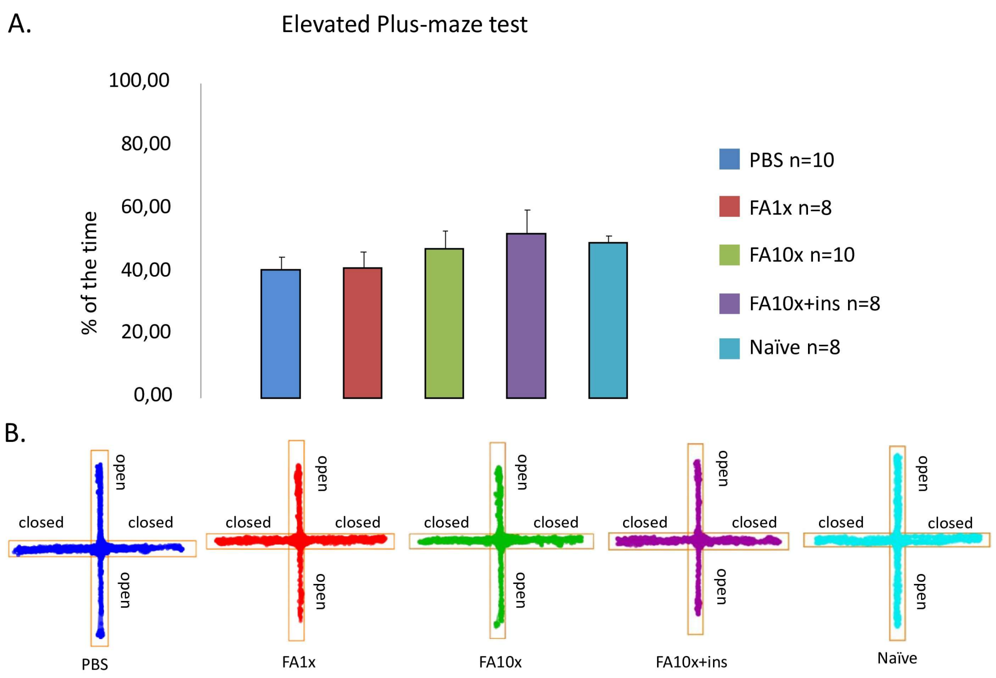

Figure S1. A. Intranasal administration procedure does not induce anxiety in the elevated plus-maze test. Results of Elevated Plus-maze test. **B**. Representative graph shows the position of the animal's centre point for the entire duration of the test. n - number of examined animals. Statistical analysis was performed using the Kruskal Wallis post-hoc Dunn's test.

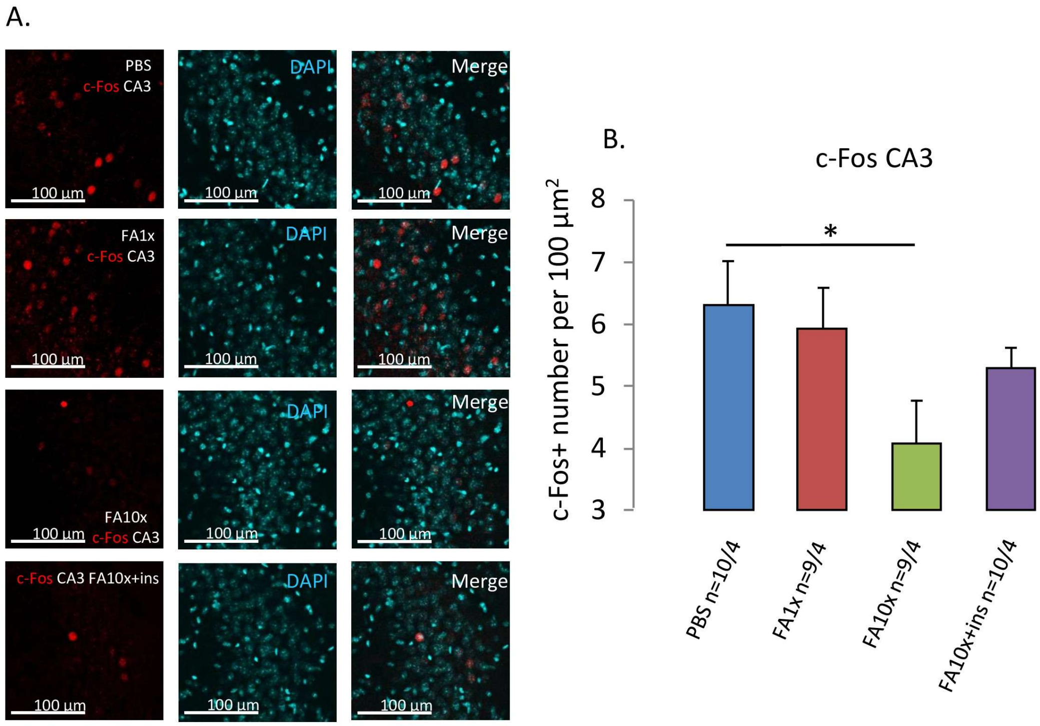

Figure S2. FA10x exposure reduces c-Fos expression in CA3 hippocampal neurons. **A**. Representative images c-fos (red), DAPI (blue) and colocation c-Fos-DAPI (merge) in CA3 neurons. Scale bar 100 µm. **B**. The results show a decrease in c-fos expression in the FA10x and FA10x+ins groups. n - number of examined slices/animals. **p*=0.04, ANOVA method and the Tukey post-hoc test.

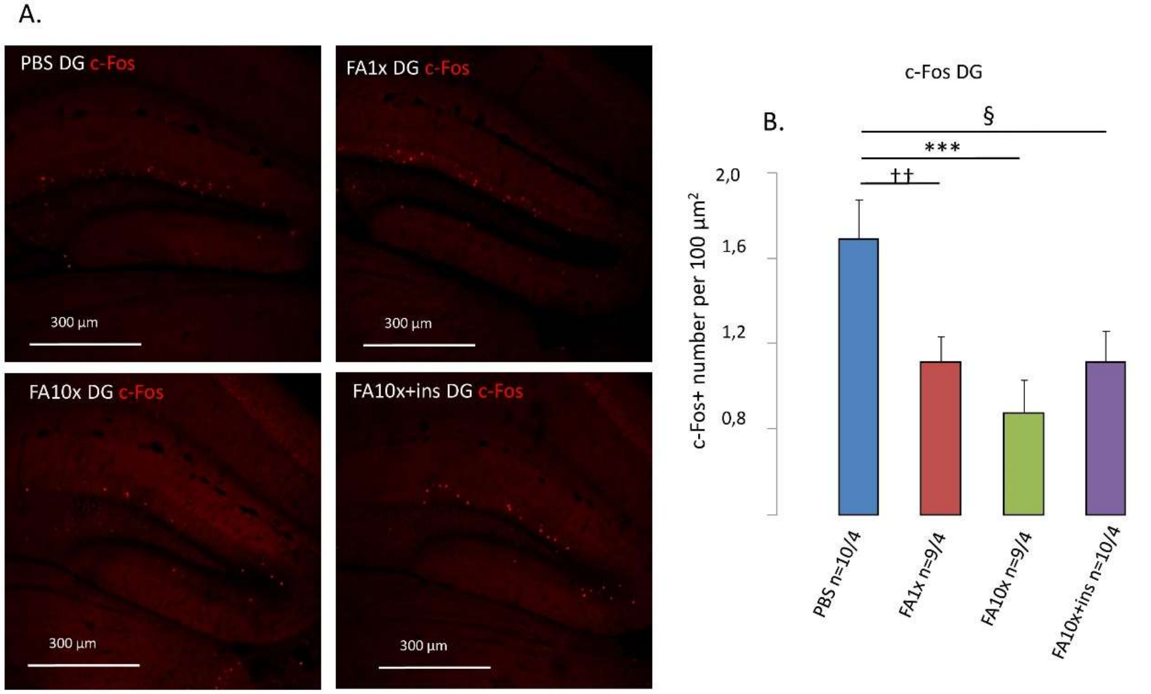

Figure S3. FA10x exposure reduces c-Fos expression in DG neurons. A. Representative images c-fos (red) in DG neurons. Scale bar 300 µm. B. The results show a decrease in c-fos expression in the FA1x, FA10x, and FA10x+ins groups. n - number of examined slices/animals. ††p=0.008, ***p<0.001, §p=0.01, ANOVA method and the Tukey post-hoc test.

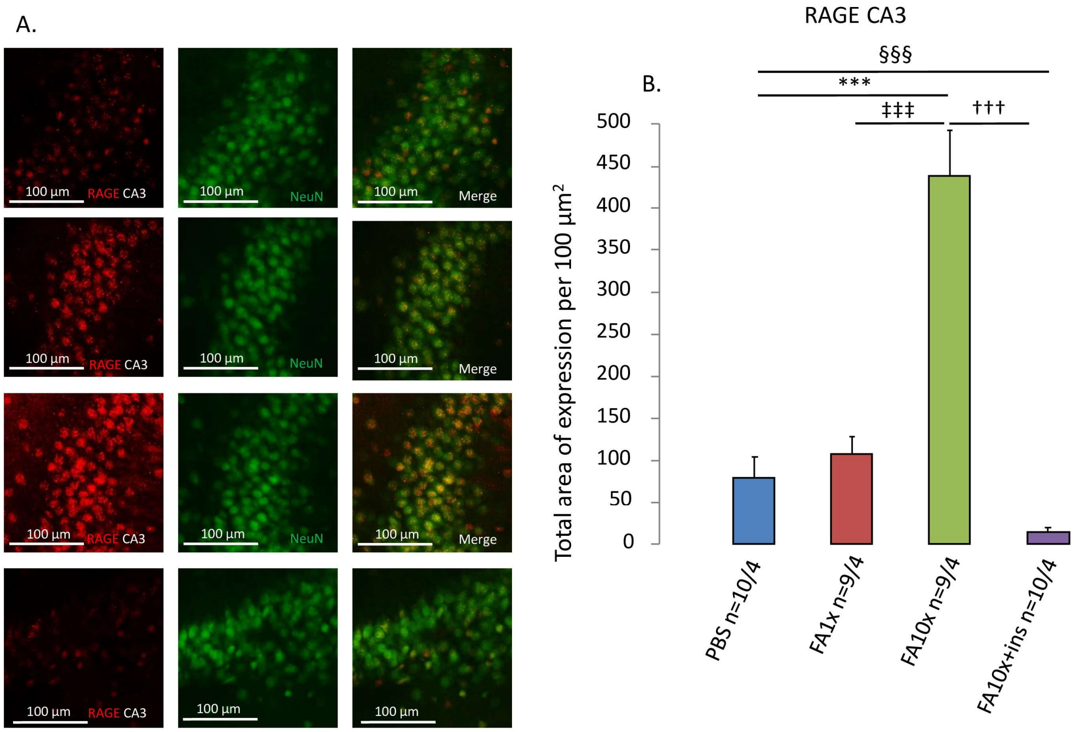

Figure S4. Intranasal formaldehyde increases RAGE expression in CA3 hippocampal neurons. **A**. Representative images of RAGE (red), NeuN (greed), colocation RAGE-NeuN (merge) in CA3 neurons. Scale bar 100 µm. **B**. The results show an increase in RAGE expression in the FA10x group, and a decrease when insulin is added. n - number of examined slices/animals. ***p<0.001, †††p<0.001, ‡‡‡p<0.001, §§§ p<0.001, ANOVA method and the Tukey post-hoc test.

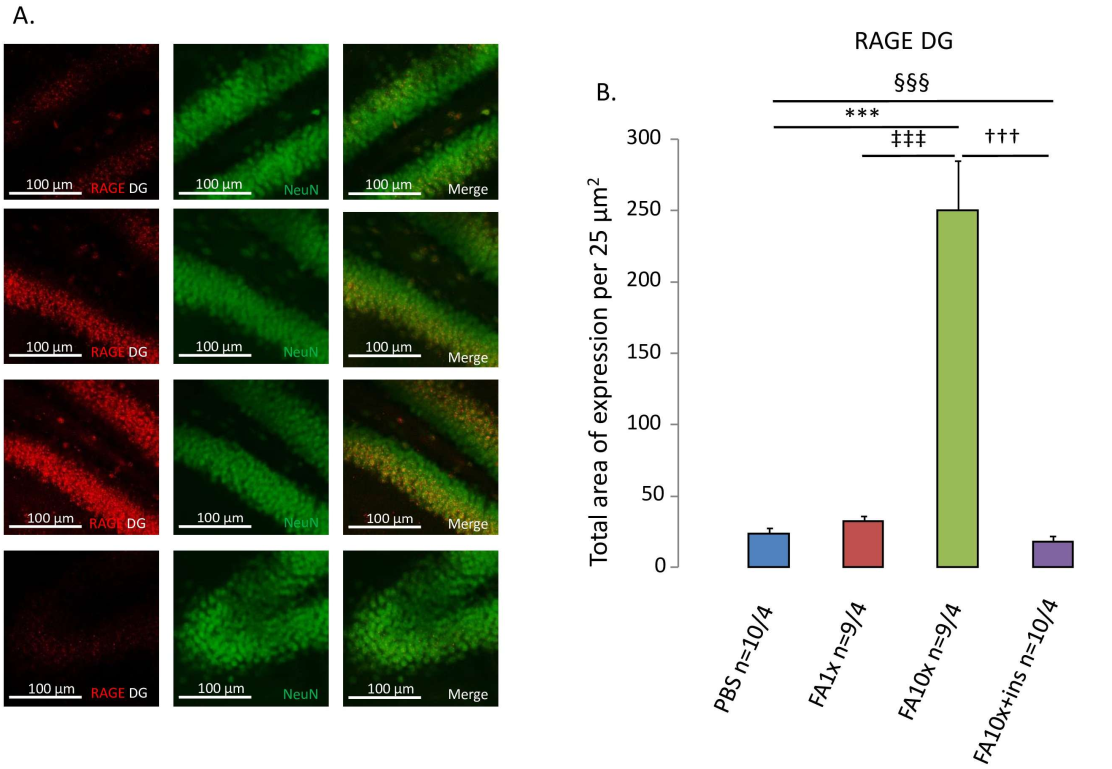

Figure S5. Intranasal formaldehyde increases RAGE expression in dentate gyrus neurons. **A**. Representative images of RAGE (red), NeuN (greed), colocation RAGE-NeuN (merge) in DG neurons. Scale bar 100 µm. * - the difference between groups. **B**. The results show an increase in RAGE expression in the FA10x group, and a decrease when insulin is added. n - number of examined slices/animals. ****p*<0.001, †††*p*<0.001, ‡‡‡*p*<0.001, §§§*p*<0.001, ANOVA method and the Tukey post-hoc test.
